# Development of a New Sr-O Parameterization to Describe the Influence of SrO on Iron-Phosphate Glass Structural Properties Using Molecular Dynamics Simulations

**DOI:** 10.3390/ma14154326

**Published:** 2021-08-03

**Authors:** Pawel Goj, Aleksandra Wajda, Pawel Stoch

**Affiliations:** Faculty of Materials Science and Ceramics, AGH University of Science and Technology, Mickiewicza 30, 30-059 Kraków, Poland; olawajda@agh.edu.pl (A.W.); pstoch@agh.edu.pl (P.S.)

**Keywords:** iron-phosphate glass, molecular dynamics, strontium, glass network

## Abstract

Iron-phosphate glasses, due to their properties, have many potential applications. One of the most promising seems to be nuclear waste immobilization. Radioactive ^90^Sr isotope is the main short-lived product of fission and, due to its high solubility, it can enter groundwater and pose a threat to the environment. On the other hand, Sr is an important element in hard tissue metabolic processes, and phosphate glasses containing Sr are considered bioactive. This study investigated the effect of SrO addition on a glass structure of nominal 30Fe_2_O_3_-70P_2_O_5_ chemical composition using classical molecular dynamics simulations. To describe the interaction between Sr-O ion pairs, new interatomic potential parameters of the Buckingham-type were developed and tested for crystalline compounds. The short-range structure of the simulated glasses is presented and is in agreement with previous experimental and theoretical studies. The simulations showed that an increase in SrO content in the glass led to phosphate network depolymerization. Analysis demonstrated that the non-network oxygen did not take part in the phosphate network depolymerization. Furthermore, strontium aggregation in the glass structure was observed to lead to the non-homogeneity of the glass network. It was demonstrated that Sr ions prefer to locate near to Fe(II), which may induce crystallization of strontium phosphates with divalent iron.

## 1. Introduction

Phosphate glasses are an important group of materials that possess many interesting features. Their properties mean that they can be applied in many different fields. Exemplary glasses containing calcium are biocompatible and can be used as bone or dental implants [1,2,3]. The addition of rare earths makes them interesting in the production of optoelectronic devices [4,5,6]. Phosphate glasses are also used as eco-fertilizers with a controlled dissolution rate [7,8,9]. However, the existence of easily hydrated P-O-P bridges leads to their corrosion, induced by water originating, for example, in a humid environment [10,11]. This effect may limit such glasses’ potential applications. The problem can be solved by the addition of Al_2_O_3_ or Fe_2_O_3_, which very strongly increase the glasses’ chemical durability. As such, a superior water-resistant material can be obtained and the glasses can be considered as matrixes in the waste immobilization process [12,13]. One of the most promising materials for this purpose is glass of the composition 60P_2_O_5_-40Fe_2_O_3_ [12,13,14]. Thus, by changing the iron-phosphate glass (IPG) composition, one can adjust the water resistance of the final material depending on the applicational purpose and obtain, e.g., a material of controlled dissolution rate.

In the case of radioactive waste immobilization, one of the major sources of nuclear waste activity is ^90^Sr isotope, which is the source of β radiation. Due to its high solubility, it can enter the groundwater from waste. Such ^90^Sr can be incorporated into bones and thus remain in the body. [12,13]. On the other hand, the similarity of strontium to calcium makes Sr an important element in hard tissue metabolic processes. Strontium plays a crucial role in new bone formation by enhancing osteoblasts’ proliferation and inhibitory effect on osteoclasts. Its addition to bioactive glasses may induce the healing of osteoporotic tissues, bone densification, or even the antibacterial properties of the implanted biomaterial [15].

The properties of IPGs depend on their structural features. Thus, the influence of strontium on the IPG structure is an interesting scientific problem.

A phosphate glass network can be described in a convenient way using Q^i^ notation. Q^i^ indicates a [PO_4_] tetrahedron connected by i common oxygen atoms with other [PO_4_] tetrahedrons. The oxygen atoms bonding two [PO_4_] tetrahedrons by P-O_B_-P bridges are called bridging oxygen atoms (O_B_), while oxygen atoms connected to only one P are called non-bridging oxygen atoms (O_NB_). The tetrahedrons can be connected to only three others [PO_4_], and the fourth oxygen atom creates a double P=O bond (O_t_) due to the penta-valency of P [16,17,18]. There may also be evidence of non-network oxygen atoms (O_NN_) that are not bonded to the phosphate network and do not take part in their depolymerization [19,20,21,22].

Iron is frequently called as an intermediate component of glass networks. This means that it can be a glass modifier and leads to network depolymerization. In favorable conditions, it can substitute phosphorous cations in the network sites and becomes a glass network former [23,24]. As a glass network former it creates an [FeO_4_] tetrahedron. On the other hand, Fe(III) is able to occupy glass network modifier positions in coordination with more than four oxygen atoms and behaves like a typical modifier of a network. It also has the possibility of changing the redox state and, as Fe(II), plays the modifier role. The role of iron in iron-phosphate glasses is the subject of a broad scientific discussion. As an example, according to [25], all Fe(III) ions are glass network formers whereas Fe(II) ions are modifiers. On the other side, there is a model proposed in [26] in which both of the iron cations are the glass network modifiers. There are also several other models in which iron plays both of the roles depending on the chemical environment of the site [22,27,28,29].

Taking all the above into consideration, this study aimed to show the influence of SrO substitution on the structural features of IPG glasses with a nominal 30Fe_2_O_3_-70P_2_O_5_ chemical composition that may be used in the waste immobilization process through classical molecular dynamics simulations.

## 2. Materials and Methods

### 2.1. Simulation Methods

This study examined glasses with the formal composition (100-x)(0.3Fe_2_O_3_-0.7P_2_O_5_)-xSrO mol. %, where x = 0, 5, 10, 15, …, 50. During the synthesis of iron-phosphate glasses, some of the iron atoms are reduced from Fe(III) to Fe(II). Since the share of Fe(II) atoms is usually in the range of 20–40% of the total iron in simulations, we used a constant value for the share equal to 30% [28,30]. It should be pointed out that the iron oxidation state depends on the glass synthesis conditions (melting temperature, atmosphere, etc.). According to this fact, glass composition may influence the glass melting conditions, and thus the redox state may change. It has been observed that an increase in modifier content the IPG glasses can lead to a decrease in the Fe(II) share [31,32]. Thus, we can assume that the Fe(II) content will decrease with the increase of the SrO in the glass.

The simulations were conducted using classical molecular dynamics with Lammps software [33]. In the simulation, approximately 50,000 ions were placed randomly in a cubic simulation box. The number of ions was set to reflect the tested glass chemical composition. The box lengths were set according to the experimental glass density. To remove the surface effects, periodic boundary conditions were applied.

The pair interactions of *i*-*j* ions were described using the Buckingham potential of the equation:

(1) $$V\left({\vec{r}}_{ij}\right)=A_{ij}\exp\left(-\frac{{\vec{r}}_{ij}}{\rho_{ij}}\right)+\frac{q_{i}q_{j}}{4\pi \varepsilon_{0}{\vec{r}}_{ij}}-C_{ij}/{\vec{r}}_{ij}^{6}$$

where ${\vec{r}}_{ij}\;$ is the distance between ions *i-j*, $\varepsilon_{0}$ is the permittivity of free space, $q_{i}q_{j}$ is the product of effective charges, and $A_{ij}$, $\rho_{ij}$, and $C_{ij}$ are parameters, given in Table 1, that were taken from the literature [19,34], except for the Sr-O potential parameters, which were fitted as described below. A short-range cut-off of 14 Å was set for the calculations of the non-Coulomb part of the potential. The Coulombic long-range forces were calculated by using the particle-mesh Ewald method [35] with a precision of 10^−6^.

To properly describe the covalent character of P-O-P and O-P-O bonds, an additional three-body term was added to the potential energy in the form proposed by Stillinger and Weber. The term parameters were taken directly from [21,36]. The simulations were conducted with the 1 fs timestep in the NVT ensemble. At the first stage, the starting temperature of 10,000 K was gradually lowered to 1600 K with relaxation steps at 5000 K and 1600 K. After 100,000 timesteps, the temperature was reduced to 300 K, at which point the system was relaxed through an additional 100,000 timesteps. A detailed description of the simulation protocol can be found in [20,21].

As an example, the glass network fragments were visualized using VESTA 3 software for three-dimensional visualization of the crystal, volumetric, and morphology data [37].

### 2.2. Development of Sr-O Interatomic Potential Parameters

According to our knowledge, there are no studies in the literature that deal with the Sr-O interaction parameters for the partial charges used in the simulations. That is why we had to determine the A_Sr-O_, ρ_Sr-O_, and C_Sr-O_ parameters of the interatomic potential. The parameters were fitted to reflect as closely as possible the crystal structure parameters of several crystalline compounds containing Sr through the use of the GULP program [38]. The set of compounds consisted of simple oxide (SrO), strontium phosphates (Sr_2_P_2_O_7_, Sr(PO_3_)_2_), and iron phosphates (SrFe(III)_3_(PO_4_)_3_O, SrFe(III)Fe(II)_2_(PO_4_)_3_, SrFe(III)_2_(P_2_O_7_)_2_). The crystalline phases were previously detected as the crystallizing compounds in the strontium-containing iron-phosphate glasses [39]. The compounds’ crystal structure parameters were taken from the Crystallography Open Database and the appropriate COD card numbers are given in Table 2. All the structures were fitted simultaneously and the relaxed fitting procedure was employed [38]. The determined parameters are collected in Table 1, whereas the reference and the simulated crystal structure parameters are summarized in Table 2.

It should be pointed out here that the fitted parameters could reproduce the crystal structure parameters of the compounds very well. The differences between the experimental and the simulated results were about a few percent or less. The Sr-O potential parameters were fitted based on the crystal structures, which were detected as crystallizing compounds in iron-phosphate glasses of similar compositions [39], and similar compounds were expected during the crystallization of the studied system. Taking into account the fact that the glass network structure should somehow reflect the possible crystallizing compounds, we assumed that the parameters would also properly describe the structural features of the simulated systems.

### 2.3. Glass Synthesis

An important point of the glass network simulations was the application of proper glass density, which in the proposed simulation protocol was maintained during the simulations. In the study, we used an experimental glass density. Therefore, at the beginning, we had to synthesize the simulated glasses. The glass synthesis was conducted with a conventional melting and quenching technique. The appropriate amounts of chemical-grade purity NH_4_H_2_PO_4_, Fe_2_O_3_, and SrCO_3_ were carefully homogenized in the planetary ball mill. To compensate for P_2_O_5_ losses during melting, approximately 20% overweight NH_4_H_2_PO_4_ was used. The batches were melted in an electric laboratory furnace in Al_2_O_3_ crucibles in an air atmosphere. The melting temperature was 1473 K and glasses were kept at this temperature for 2 h. The melts were vitrified by casting onto steel plates. The synthesis procedure is known to give glass compositions close to the limit of about one percent, and Al_2_O_3_ contamination is below 1% with an Fe(II) content of about 30% of the total iron. More details can be found in [22,28,40,41].

The glasses density was measured using the Archimedes method and deionized water was used as the immersed liquid. The density was measured for five pieces of glass from every composition and mean values and mean standard deviations were obtained.

## 3. Results and Discussion

**Figure 1:**
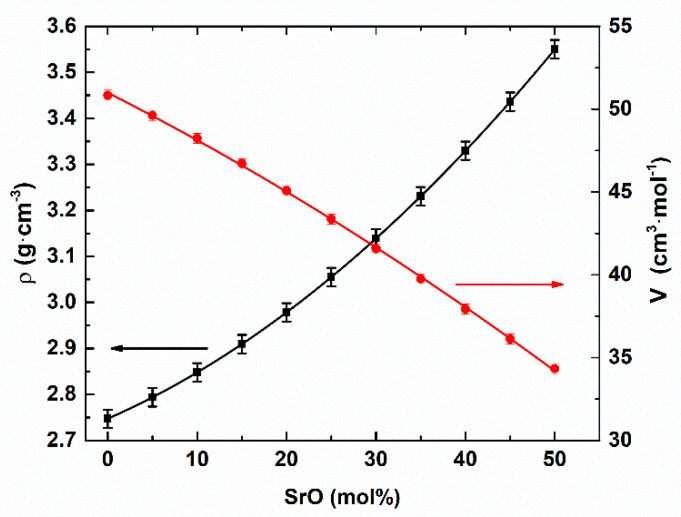
Density and molar volume dependencies on the glass composition.

The experimentally determined glass densities and corresponding molar volumes are presented in Figure 1. The molar volume was calculated according to the formula V = M/ρ, where ρ is the glass density and M is the molar mass of the glass.

The glass density increased according to the formula ρ(x) = 1.5·10^−4^x^2^ + 0.0856x + 2.7472 (g/cm^3^). More information concerning the glass network changes can help determine the molar volume. The molar volume decreased according to the relation V(x) = −0.0012x^2^ −0.2747x + 51.0002 (cm^3^/mol). The decrease indicated that the glass network had become more compacted. That may have been related to the placing of the Sr atoms in the open voids of the glass network; in this way, the network free space would have decreased. This strongly suggests the modifier role of strontium in the glass network.

**Figure 2:**
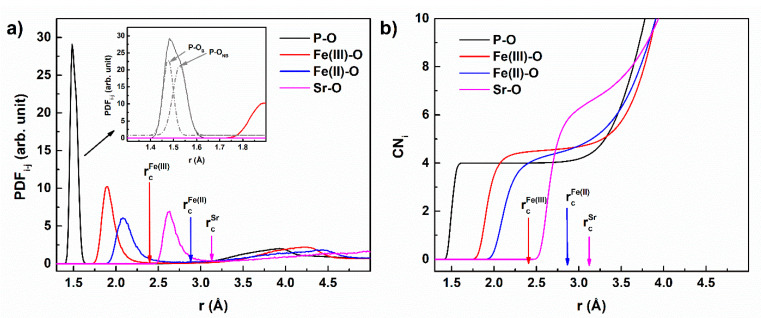
The P-O, Fe(III)-O, Fe(II)-O, and Sr-O pair distribution functions (PDFs) for 5 mol% of SrO (**a**) and their corresponding average coordination numbers (CNs) analysis (**b**) as a function of distance.

The short-range order in the glasses can be described by the partial distribution function (PDF). Exemplary PDF curves for P-O, Fe(III)-O, Fe(II)-O, and Sr-O pairs for x = 5 glass are presented in Figure 2a.

**Figure 3:**
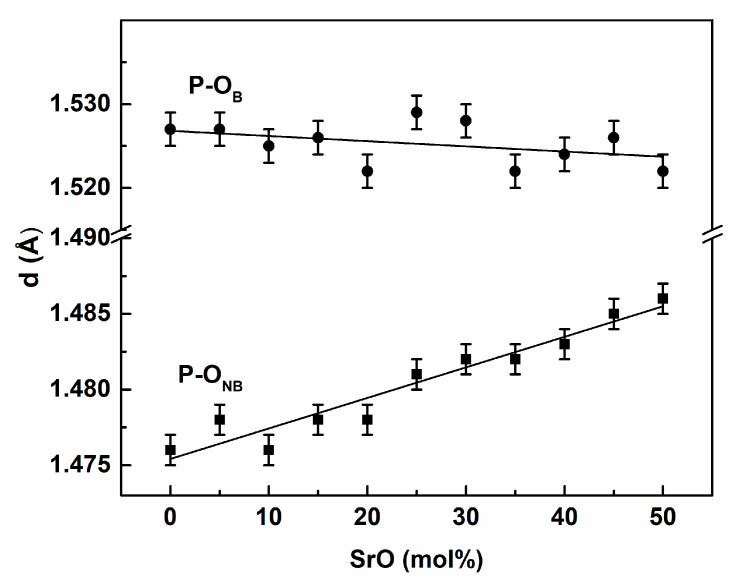
Dependence of P-O_B_ and P-O_NB_ distances on composition.

As would be expected for amorphous systems, the curves were characterized by a single well-developed peak which as the effect of the short-range order in the closest coordination shell. The further peaks were considerably less intense, which proved the glassy character of the simulated systems. For the rest of the tested materials, the obtained curves were very similar. It should be noted that the first PDF peak for the P-O pair was composed of two peaks (as shown in Figure 2a), which were attributed to two main P-O bond lengths, depending on the role of oxygen in the [PO_4_] tetrahedron. The first peak of the lower P-O length was attributed to shorter P-O_t_ or P-O_NB_ bonds with the maximum at ca. 1.475 Å. This corresponds fairly well with the literature data, where the bond length is in the range of 1.43–1.49 Å [20,21,22,41,42,43,44,45]. The distance increased linearly with the SrO content, as presented in Figure 3 (P-O_NB_ curve). This increase may be attributed to the increased content of P-O_NB_ bonds compared to P-O_t_ bonds. P-O_t_ bonds are shorter than P-O_NB_ bonds [22,41,45]. Thus, with the increase of the P-O_NB_ content, the position of the PDF curve component moves toward the higher lengths. The effect may also indicate that Sr ions prefer to break double P-O_t_ bonds and transform them into P-O_NB_-Sr joinings, instead of disrupting P-O_B_-P bridges.

The P-O_B_ bond length was greater and the corresponding component of the first PDF peak was located for the longer distances (Figure 2.). The position of the peak was between 1.52 and 1.53 Å and was almost constant (Figure 3). The P-O_B_ distances followed previous MD results indicating ca. 1.51–1.64 Å [17,19,21,29,41,42,43,45,46,47,48]. Nevertheless, it should be noted that the MD values are lower than the values from ab initio simulations, which are closer to 1.6 Å [22,29,41,45]. This may be an effect of the simulation model and the P-O interatomic potential parameters used.

The integration of the PDF curve gives the dependence of the coordination number of the given central atom on the distance. Therefore, the integration gives the average coordination number (CN) of P to oxygen as a function of the distance from the central P atom. The obtained CN functions are presented in Figure 2b. The CN_P_ appeared as a step-like curve that is characteristic of a glass network species, and the P atoms took, in a step-like manner, a constant coordination number of 4 in relation to oxygen; further, the value was independent of the assumed cut-off radius in a wide region from above 1.6 Å up to ca. 2.6 Å. This confirmed that all the phosphorous atoms were placed in the middle of the oxygen tetrahedrons [PO_4_]. Above 2.6 Å the second coordination sphere started.

**Figure 4:**
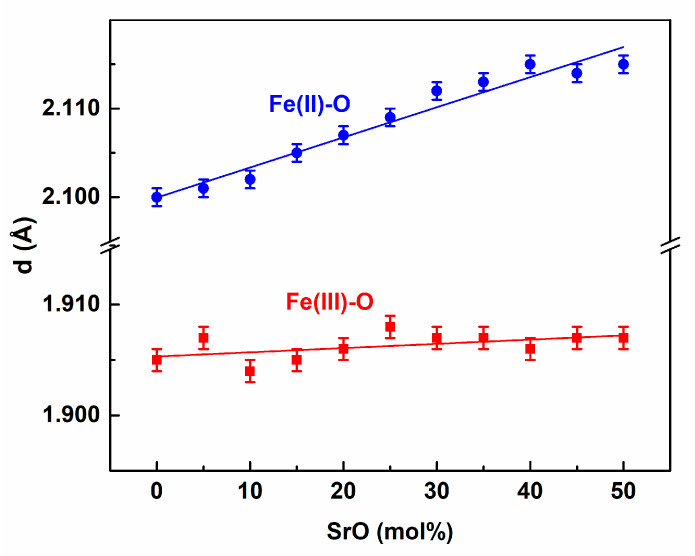
The Fe(III)-O, and Fe(II)-O distances as a function of the glass composition.

The longer bond length created an Fe(III)-O pair. Therefore, the maximum of the PDF curve lay at the higher lengths (Figure 2). The position of the maximum was about 1.9 Å and slightly increased with the SrO content, as presented in Figure 4. The value was in accordance with earlier studies of IPGs where the distance was in the range of 1.87–1.91 Å [19,21,26,29,45,49].

**Figure 5:**
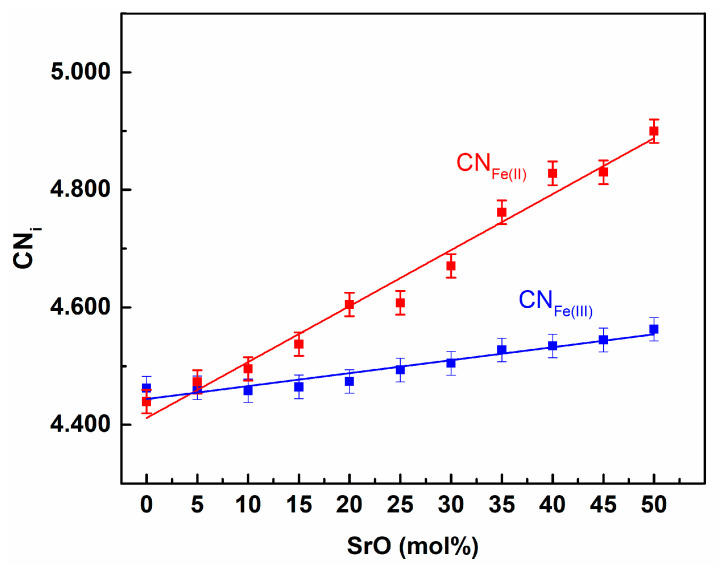
Iron coordination numbers for oxygen vs. SrO content in the glass.

The CN curve for the Fe(III)-O (Figure 2b) also showed step-like behavior. Nevertheless, in this case, the step was not flat and increased with the distance. Therefore, the coordination number depended on the value of the cut-off radius taken. In the further analysis, the cut-off radius was defined as the distance which had the minimum value between the first and the second peaks on the corresponding PDF curve. For Fe(III)-O the minimum value was at 2.4 Å, and this value was used as the cut-off radius. Thus, the average coordination numbers for iron in relation to oxygen could be obtained and are shown in Figure 5.

**Figure 6:**
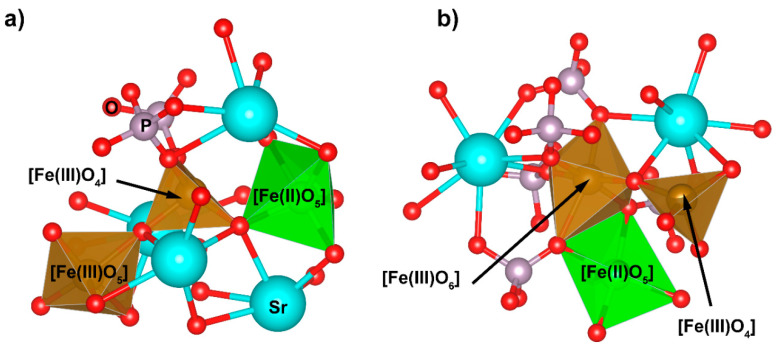
Exemplary bipyramidal (**a**) and octahedral (**b**) Fe(III) sites for x = 30 glass.

It can be seen that the CN number for Fe(III) linearly increased with the SrO content in the glass and was about 4.5. This is in good agreement with earlier MD simulations [19,20,21]. In iron-phosphate glasses, iron atoms have a dual role depending on the crystal–chemical surroundings. They may substitute phosphorous atoms in their network positions in a tetrahedral coordination or may function as glass network modifiers with the higher coordination numbers. In the simulated glasses, the mean coordination of ca. 4.5 was realized by about 52% of the Fe(III) in tetrahedral positions, 45% in distorted bi-pyramidal sites (CN = 5), and only 3% in octahedral sites. Exemplary Fe(III) surroundings are presented in Figure 6.

Thus, we assumed that about half of the iron Fe(III) atoms occupied glass network positions. Nevertheless, it should be noted that this assumption was based on the value of the cut-off radius used and the geometry of the sites. A much more precise determination of the coordination number could be obtained based on bond order analysis, in which only interacting atoms are taken into account. It has been shown that in iron-aluminum-phosphate glasses, some oxygen atoms lying closer, e.g., as in aluminum, do not create a chemical bond, whereas those lying further away do create a bond [22,41]. Such an analysis is beyond the capacities of classical methods and can only be done by utilizing ab initio simulations.

Similar behavior to that of Fe(III) was evidenced for Fe(II). However, the CN function was less step-like, which was the reason for the lower short-range ordering. In this case, the distances were longer and the coordination number was also higher. This was a reason for the Fe(II) atoms‘ greater size and lower contribution to the directional, covalent-character interaction in the Fe(II)-O chemical bond compared to Fe(III)-O [22,29].

**Figure 7:**
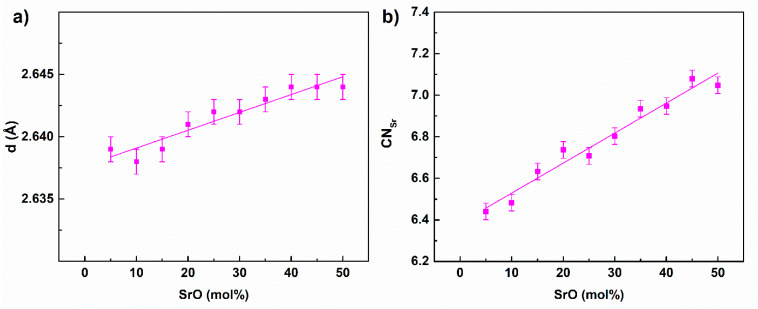
The Sr-O distance (**a**) and coordination number (**b**) as a function of the glass composition.

The longest distance was the Sr-O distance, which increased with SrO content in the glass (Figure 7a), and the mean coordination number for oxygen also increased from ca. 6.4 Å to ca. 7 Å (Figure 7b). It should be noted that in the fitted crystal structures strontium atoms were in coordination 6 or 7 in relation to oxygen, with the mean bond length being between ca. 2.56 and ca. 2.69 Å, which is in agreement with previous results. The Sr-O bond distance was comparable with but higher than that of Ca-O, which is about 2.43 Å in similar IPGs [20]. The difference seems to be reasonable taking into account the fact that strontium is larger than calcium.

We can make the general conclusion that the increase of SrO content increased the mean coordination numbers for oxygen for both forms of iron and strontium. The increase was related to the extension of the mean distances from oxygen. In all the cases, this was a linear growth that confirmed the correlation between the distance and the coordination number. Additionally, we may assume that the chemical bond lengths followed the trend. Similar behavior has been observed for calcium atoms in IPGs, although the calcium CN was higher by about 0.3 [20].

**Figure 8:**
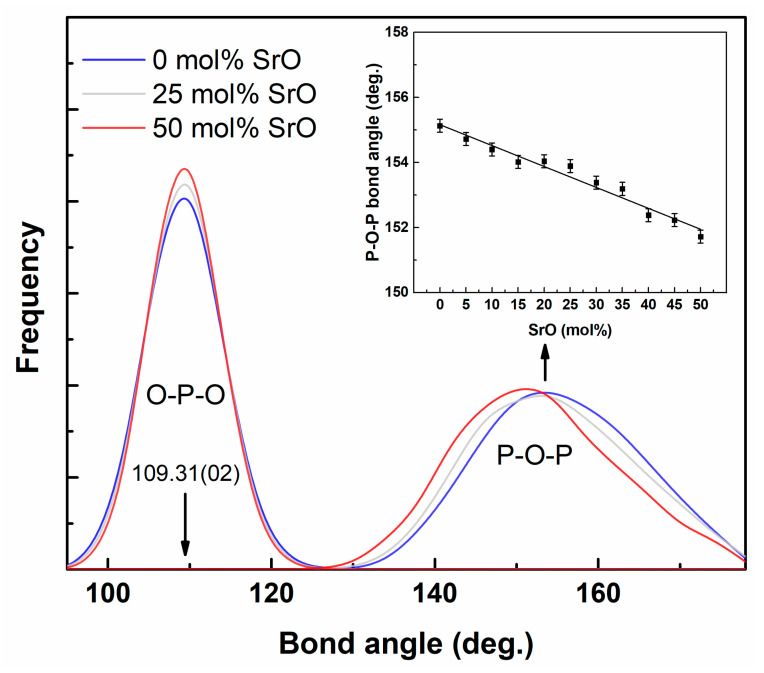
The angular distributions of O-P-O and P-O-P bonds for the glasses.

In the simulated glasses, P_2_O_5_ was the only pure network oxide. Therefore, in the further description of the glass network, we focus mostly on this cation. The important parameters describing the network are the O-P-O and P-O-P angle distribution functions (ADFs). The ADFs for the simulated glasses are presented in Figure 8.

The O-P-O distribution describes the bond angles in [PO_4_] tetrahedrons. In an ideal tetrahedron, the distribution would have a maximum for the angle 109.47^0^, and a very similar maximum was observed in this study. The position of the maximum was independent of the glass composition. The intensity of the peak slightly increased with the SrO content and followed a very slight decrease of the half-width. This was an effect of the decrease in the number of O_t_ oxygen atoms and their transformation to O_nb_. The difference in bond length between P-O_NB_ and P-O_B_ was lower compared to P-O_t_. Thus, the [PO_4_] tetrahedron was less distorted and the corresponding ADF was narrower. The P-O-P angle is the angle at which [PO_4_] tetrahedrons are joined by each other. In this case, the distribution was wider due to a much higher level of randomness. The position of the maximum linearly decreased with the SrO content in the glass (Figure 8—inset), which may have been an effect of the phosphate chains’ shortening.

**Figure 9:**
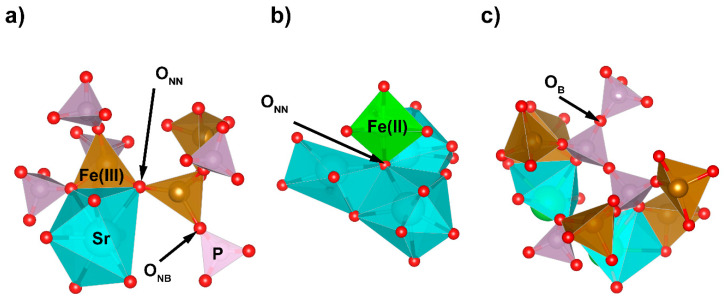
Examples of atom clusters with O_NB_ (**a**); O_NN_ (**b**); and O_B_ (**c**) atoms.

Glass network connectivity (NC) is another important parameter that can be used in the description of a glass network. The NC is defined as the number of bridging oxygen atoms per glass network-forming cation [24]. To initially determine the, one must establish the distribution of different oxygen types in the glass. In the studied system, there were four different oxygen atom types. There were terminal oxygen atoms (O_T_), which create double P=O bonds; non-bridging atoms (O_NB_), which are the atoms that create linkages for the P-O-Fe/Sr bonds; bridging oxygen atoms (O_B_), which form P-O-P bridges; and non-network oxygen atoms (O_NN_), which are not connected with phosphorous. Examples of atom clusters where the oxygen atom types are present are shown in Figure 9.

**Figure 10:**
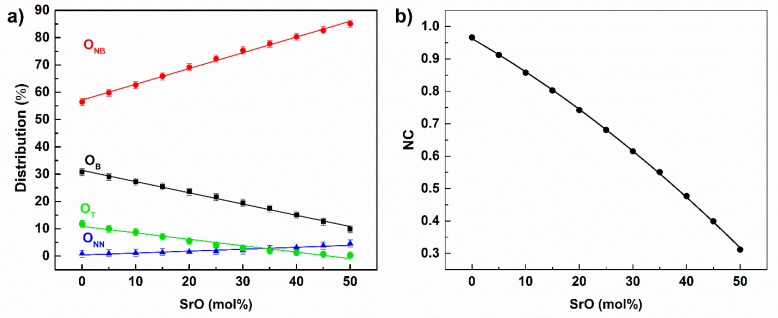
Dependence of O_B_, O_NB_, O_T_, O_NN_ (**a**), and network connectivity (**b**) on glass composition.

The distribution of the oxygen atom types as a function of the glass composition is shown in Figure 10a.

It can be seen that the number of O_B_ oxygen atoms decreased with the SrO content, and the decrease accompanied an increase in the numbers of O_NB_ and O_NN_ oxygen atoms. The O_B_ decrease together with the increase of O_NB_ demonstrated the glass network’s gradual depolymerization due to the increase of the SrO content in the glass. This was confirmed by the decrease of the NC parameter, which followed the linear trend of O_B_ changes. Based on the calculated O_B_ values, the NC parameter decreased from 0.97 for x = 0 to 0.31 for x = 50 glass (Figure 10b). Thus, the phosphate network was almost continuous only for the base glass where the NC was close to 1.

**Figure 11:**
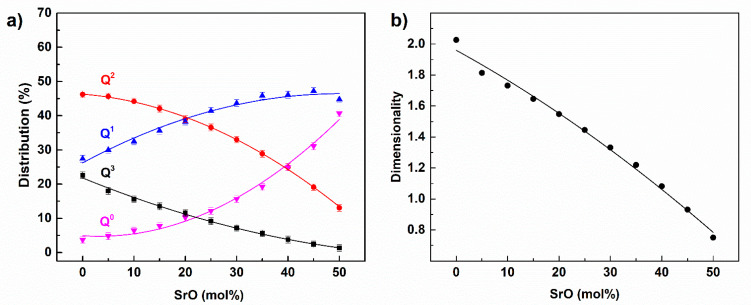
Distribution of Q^i^ structural units in the simulated glasses (**a**) and the dimensionality of the glass network (**b**).

The distribution of different Q^i^ structural units in the simulated glasses and the dimensionality of the glass network, defined as the mean i parameter of the Q^i^ structural units, are given in Figure 11.

**Figure 12:**
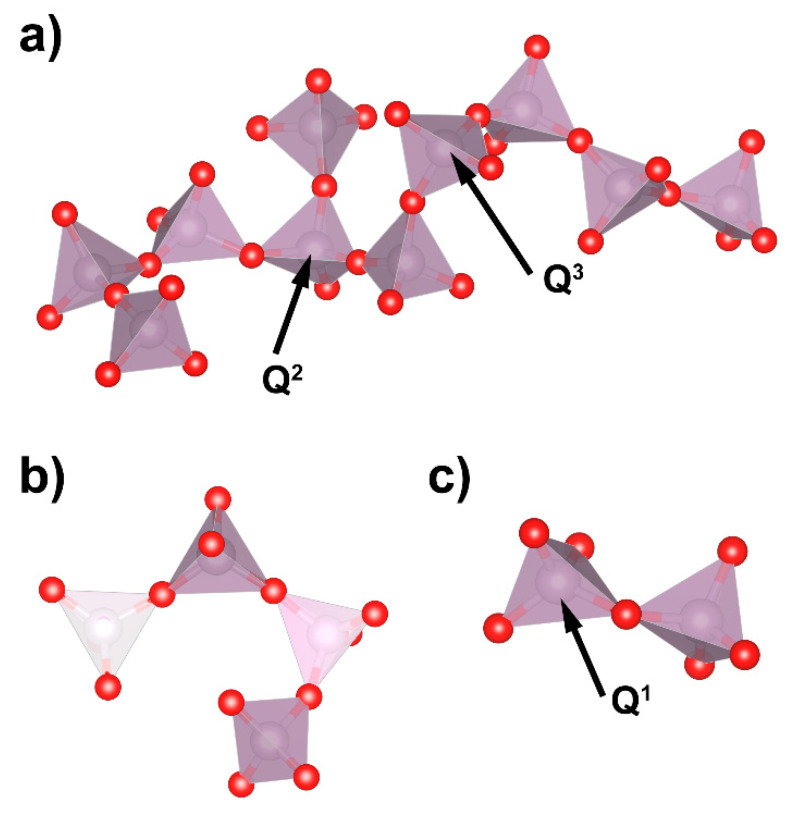
Exemplary phosphate network elements for x = 0 (**a**), x = 25 (**b**), and x = 50 (**c**) glasses.

Exemplary phosphate network elements for the selected glasses are presented in Figure 12.

The base glass was built mostly of Q^2^ with some comparable additions of Q^1^ and Q^3^ structural units. Thus, the phosphate network was composed of long chains that were joined to each other by Q^3^ units and terminated by Q^1^ units (Figure 12a). The addition of SrO led at first to a decrease in the number of Q^3^ units. Therefore, the connected chains were being separated. Then, for the higher SrO content, the decrease in Q^2^ units became more rapid and, in its place, formation of Q^1^ and Q^0^ occurred. Thus, in the middle range of SrO in the glass, the long chains were being shortened and even separated Q^0^ units were distinguished (Figure 12b). The high modifier-content glass was built of very short chains, like Q^1^ dimers and separated Q^0^ units (Figure 12c). This followed the decrease in the glass network dimensionality (Figure 11b), which was the average *<i>* value of the Q*^i^* unit distribution [24,41]. Initially, the glass created a 2D phosphate network, in which the average dimensionality was decreased to one-dimensional chains for x = 40.

The glass network dimensionality could also be simply predicted based on the O/P ratio. In the case of the pure P_2_O_5,_ the O/P = 2.5 and <*i*> = 3. The parameter decreased linearly with the ratio through O/P = 3, <*i*> = 2 (metaphosphate) and O/P = 3.5, <*i*> = 1 (pyrophosphate) until O/P = 4, <*i*> = 0 (orthophosphate) [17]. According to the ratio, the base glass (x = 0) had O/P = 3.14, which resulted in <*i*> = 1.71. The value was lower compared to the simulated <*i*> of 2.02 (Figure 11b). For the glasses, we assumed that 30% of the Fe(III) atoms were reduced to Fe(II), which would have been connected with some oxygen loss. After considering this effect, the O/P ratio was judged to equal 3.08 and the corresponding <*i*> 1.84. Additionally, in the system, there were also O_NN_ oxygen atoms that did not take part in the network depolymerization. Taking into account the O_NN_ oxygen atoms led to an <*i*> value of 1.92, which was much closer to the simulated <*i*> value of 2.02. A similar result was found for all the simulated glasses. For example, for x = 25, considering only the iron reduction led to <*i*> = 1.37, whereas including O_NN_ atoms led to <i> = 1.48. The value was close to the simulated value of <*i*> = 1.45. Thus, comparing the experimentally obtained dimensionality with the value predicted based on the glass stoichiometry provided the possibility of estimating the quantity of O_NN_ oxygen, as proposed in [28,41].

**Figure 13:**
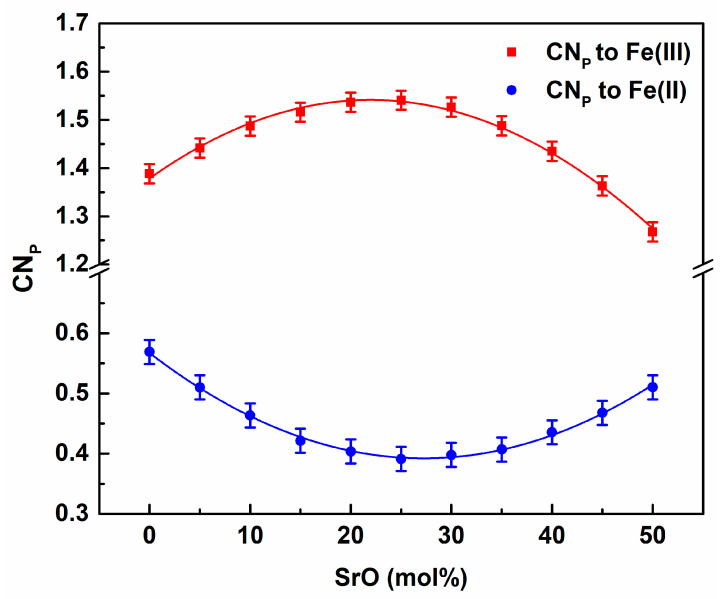
Compositional dependence of the coordination numbers P for Fe(III) and Fe(II).

Interesting information was gained from the analysis of the average coordination number of P for Fe(III) and Fe(II). The dependence on the SrO content in the glass is presented in Figure 13.

**Figure 14:**
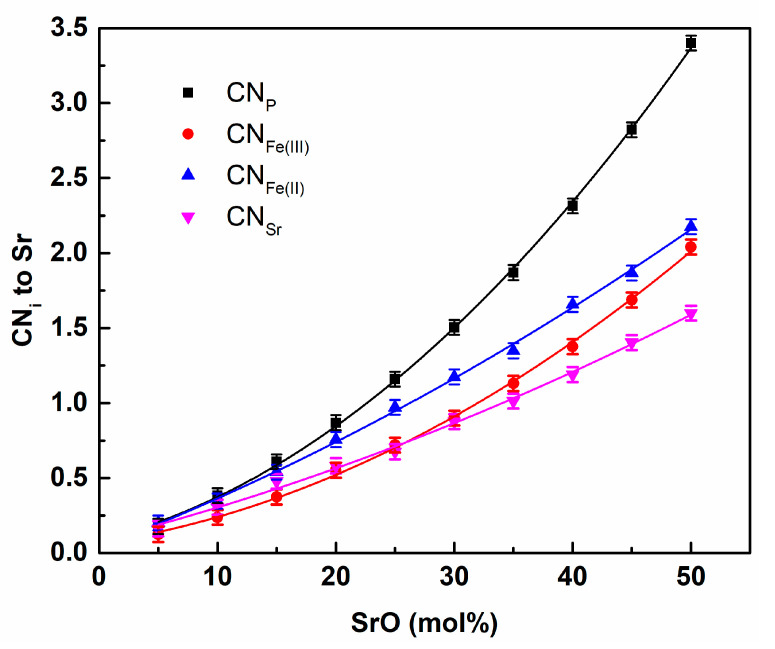
Compositional dependence of the coordination numbers for Ca for P, Fe(II), Fe(III), and Sr.

The number of Fe(III) around P initially increased and achieved a maximum for about x = 0.25 before decreasing. The curve for Fe(II) demonstrates the opposite tendency. It suggests that, for low Sr contents, Fe(II) atoms could be removed from the phosphorous surroundings. This shows that strontium may prefer to be located near Fe(II) sites in a network. On the other hand, the withdrawing of Fe(II) from P was compensated for by the affinity of Fe(III). This was confirmed by analyzing coordination numbers for Sr for metal cations. The compositional dependence is shown in Figure 14.

It can be clearly seen that the coordination numbers for all the cations increased with increasing SrO content. The fastest increase was for Sr around P, which showed a preference for strontium to be located around [PO_4_] tetrahedra. This is quite natural and has been previously detected for sodium and calcium [20,21]. Despite the fact that Fe(II) only comprised 30% of the total iron in the glass structure, strontium had a higher preference for locations near Fe(II) than Fe (III).

This supports the idea of Sr’s affinity for Fe(II). It should be pointed out that such behavior may lead to a preference for the crystallization of compounds containing Sr and Fe(II). This was observed experimentally, for which in the comparable Sr-containing IPGs the major crystalline compound was SrFe(II)_2_P_2_O_7_ [39].

**Figure 15:**
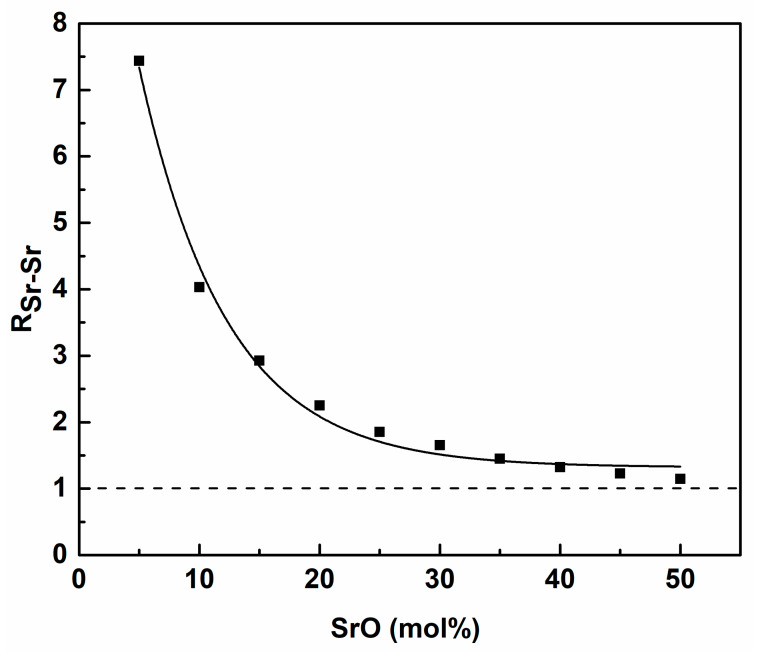
Dependence of the Sr-Sr aggregation parameter (R_sr-sr_) of the glass composition.

The calculated values of the coordination numbers of Sr for Sr were used to obtain the aggregation parameter (R_Sr-Sr_), as defined in [50,51,52]. The parameter was a simple ratio of the coordination number to a random homogenous distribution. A value over 1 indicated ions clustering. The estimated values of the aggregation parameter are shown in Figure 15.

The values of the parameter show that, with low Sr content, very strong aggregation of the atoms could be observed. This led to the formation of Sr-rich clusters and the inhomogeneity of the glass structure. The increase of SrO content led to an increase of the regions and, in this way, the whole glass network became more homogenous. A similar effect has been observed for Ca [20]. However, in the case of Ca, the parameter value was lower and, for the low modifier, the contents were about twice the Ca value. Thus, we judged that the aggregation of Sr was stronger than that of Ca.

In light of the obtained results, it can be observed that the glass network was inhomogeneous, with regions enriched and depleted in specific species alongside more ordered regions, like in Lebedev crystalline theory, which interlaces with more disordered regions of Zachariasen–Waren theory. Thus, the network was close to the model domain proposed by Görlich [53,54].

## 4. Conclusions

New interatomic potential parameters for Sr-O interaction were developed and tested for crystalline compounds.

The influence of increasing SrO content on the structural properties of 30Fe_2_O_3_-70P_2_O_5_ glasses was tested using classical molecular dynamics. The short-range structure results were in good agreement with previous experimental and theoretical studies.

The glasses with low SrO contents were built of chains that can have branches and form three-dimensional structures. The increase in SrO content in the glass led to phosphate network depolymerization, which was characterized by decreases in glass connectivity and dimensionality parameters.

It was observed that non-network oxygen atoms existed in the studied system that did not take part in the phosphate network depolymerization. The quantity of the atoms can be estimated through a comparison of the experimental dimensionality parameter with the value predicted based on the glass stoichiometry.

It was detected that Sr ions prefer to be located near to Fe(II), which can induce crystallization of strontium phosphates with divalent iron.

Evidence of strontium aggregation was found, and it was the most intense for low SrO contents. This can lead to the inhomogeneity of the glass network with the formation of Sr-rich and Sr-depleted regions.

## Figures and Tables

**Table 1:** Values of the Buckingham potential parameters of the *i*-*j* pair [19,34].

Pair (*i*-*j*)	q*_i_* (e)	A*_ij_* (eV)	q*_ij_* (Å)	$C_{ij}(\mathrm{eV}\cdot {Å}^{6})$
Fe(II)-O	1.2	11,777	0.2071	21.642
Fe(III)-O	1.8	19,952	0.1825	4.6583
O-O	−1.2	1844	0.3436	192.58
P-O	3.0	27,722	0.1819	86.860
Ca-O	1.2	131,400	0.1875	60.0
Sr-O	1.2	433,269,870	0.120561	78.25863

**Table 2:** Reference and fitted crystal structure parameters.

Cell Parameter	Simulation	Reference	Difference (%)
SrO (space group Fm-3; COD-1011328)			
V (Å^3^)	140.181	132.963	5.43
a (Å)	5.195	5.104	1.78
b (Å)	5.195	5.104	1.78
c (Å)	5.195	5.104	1.78
α (^o^)	90.000	90.000	
β (^o^)	90.000	90.000	
γ (^o^)	90.000	90.000	
Sr_2_P_2_O_7_ (space group Pmmm; COD-1528537)			
V (Å^3^)	637.422	634.429	0.47
a (Å)	8.855	8.870	−0.17
b (Å)	13.306	13.270	0.29
c (Å)	5.409	5.390	0.36
α (^o^)	90.000	90.000	
β (^o^)	90.000	90.000	
γ (^o^)	90.000	90.000	
Sr(PO_3_)_2_ (space group P_1_21/c1; COD-2008912)			
V (Å^3^)	960.913	998.300	−3.75
a (Å)	7.073	7.209	−1.89
b (Å)	7.808	7.953	−1.82
c (Å)	17.399	17.414	−0.09
α (^o^)	90.000	90.000	
β (^o^)	89.772	90.640	−0.96
γ (^o^)	90.000	90.000	
SrFe_3_(PO_4_)_3_O (space group P2/m; COD-1531869)			
V (Å^3^)	491.646	486.588	1.04
a (Å)	7.571	7.540	0.41
b (Å)	6.357	6.348	0.15
c (Å)	10.381	10.316	0.63
α (^o^)	90.000	90.000	
β (^o^)	100.261	99.740	0.52
γ (^o^)	90.000	90.000	
SrFe_3_(PO_4_)_3_ (space group Imma; COD-1521446)			
V (Å^3^)	953.990	916.270	4.12
a (Å)	10.396	10.452	−0.54
b (Å)	13.712	13.429	2.11
c (Å)	6.692	6.528	2.52
α (^o^)	90.000	90.000	
β (^o^)	90.000	90.000	
γ (^o^)	90.000	90.000	
SrFe_2_(P_2_O_7_)_2_ (space group P-1; COD-2003248)			
V (Å^3^)	251.857	255.119	−1.28
a (Å)	4.784	4.795	−0.23
b (Å)	7.155	7.108	0.66
c (Å)	7.758	7.830	−0.91
α (^o^)	88.072	89.830	−1.96
β (^o^)	89.162	87.590	1.80
γ (^o^)	71.615	73.110	−2.04

## Data Availability

The data presented in this study are available on request from the corresponding author.
